# Factors Influencing Interns' Selection of Postgraduate Residency Programs in the United Arab Emirates

**DOI:** 10.7759/cureus.95161

**Published:** 2025-10-22

**Authors:** Khaled Aldahmani, Jayadevan Sreedharan, Salah Eldin Kassab

**Affiliations:** 1 Department of Internal Medicine, Tawam Hospital - SEHA, Pure Health, Al Ain, ARE; 2 Department of Medicine, College of Medicine and Health Sciences, United Arab Emirates University, Al Ain, ARE; 3 Department of Epidemiology and Biostatistics, College of Medicine and Thumbay Institute of Population Health, Gulf Medical University, Ajman, ARE; 4 Department of Medical Education, College of Medicine, Dubai Medical University, Dubai, ARE

**Keywords:** career choices, influencing factors, internship, medical education, residency training

## Abstract

Introduction: Choosing a residency program is a critical decision for medical interns, shaping their future careers. In the United Arab Emirates (UAE), little is known about the factors influencing interns' selection of their residency training program of choice.

Objectives: To evaluate the different factors that medical interns consider when selecting their residency training program of choice.

Methods: A cross-sectional study was conducted using online questionnaires from May to June 2024. All interns registered in the Department of Health and Emirates Health Services for the 2023-2024 year were invited. The survey assessed demographics, program preferences, and key factors affecting residency selection.

Results: Out of 300 invited interns, 170 responded (56.7%). The majority were female (n = 112, 65.9%). Interns represented 18 nationalities; the majority came from three countries: UAE (n = 72, 42.4%), India (n = 21, 12.4%), and Iraq (n = 13, 7.6%). The three most preferred residency choices were general medicine (n = 23, 15.5%), pediatrics (n = 19, 12.8%), and emergency medicine (n = 19, 12.8%). The top factors influencing residency selection were interest in specialty (n = 117, 70%), opportunities for involvement in patient care (n = 117, 68.8%), performing procedures (n = 113, 66.5%), and professional development (n = 107, 62.9%). About 96% (n = 163) believed that program accreditation is essential, with the National Institute of Health Specialties (n = 60, 37%) and Accreditation Council for Graduate Medical Education - International (n = 44, 27.1%) being the two most valued accreditation associations.

Conclusion: This study highlights the most preferred residency programs among the interns and identifies general and discipline-specific factors influencing interns' selection, providing insights for stakeholders to enhance postgraduate residency training and align with interns' expectations.

## Introduction

Physicians undergo different stages of training to ensure they are competent and able to provide specialty services to the community [1]. Selecting a residency program is a high-stakes decision for interns, carrying significant implications for their future careers. This decision-making process is multifaceted, involving various factors such as program quality, personal interests and work-life balance considerations. Importantly, these factors significantly evolve during the internship year as the interns go through their scheduled rotations to the different clinical departments and the direct involvement in patient care and management. In a large metanalysis, the top five factors impacting subspeciality program selection were academic interest, competencies, flexible work schedule, patient service involvement, and the impact of medical teachers or mentors [2]. Similarly, a questionnaire-based study in India including 368 medical students and interns highlighted personal and professional growth as well as personal satisfaction as important motivating factors influencing residency program selection [3].

In United Arab Emirates (UAE), previous studies showed that surgery and internal medicine were the two most preferred specialties among medical students [4,5]. Intellectual fulfillment, work-life balance, possessing the necessary talent and potentials for specialty advancement were the main factors affecting the residency program selection [4,5]. However, these studies have primarily focused on medical students. Also, there has been a significant advancement in medical education in UAE since then with progressive increase in the number of different residency and fellowship training programs across the country [6,7]. In addition, the National Institute of Healthcare Specialties (NIHS), an important milestone in the country’s medial education and healthcare system, was launched in 2021 [8].

The primary aim of this study is to evaluate the different factors that medical interns consider when selecting their residency training program of choice. The secondary aims are to explore factors influencing choices among different medical and surgical programs and to identify interns’ most valued accreditation association/board.

## Materials and methods

All interns enrolled in the Department of Health in Abu Dhabi and Dubai, as well as those under the Ministry of Health through Emirates Health Services during the academic year of 2023-2024, were invited to participate. A cross-sectional design was adopted, and an electronic survey using the Microsoft platform was distributed between May and June 2024. A unique survey link was distributed to each participating institution. Responses were cross-checked using demographic data to minimize duplicate entries. Interns who did not provide consent for participation were excluded.

We used a structured survey questionnaire, which was adapted from a similar study in India [3]. After modifying a few items to align with the UAE context, the content validity of the questionnaire was re-evaluated. The revised items were reviewed by two medical education experts to ensure relevance and clarity. Subsequently, a pilot test was conducted with 19 interns from Tawam Hospital (Appendix 1) to assess the clarity, comprehension, and appropriateness of the survey items.

The survey included 15 questions over three sections. The first section covered the demographics and background related to medical training. The second part evaluated residency training program interest and 15 influencing factors. Interns were also asked to add additional factors and share their plans if not matched. The third part assessed the importance of accreditation on the decision to select their program of interest. Interns were asked to rank the top three accrediting bodies among the following: NIHS, Accreditation Council for Graduate Medical Education- International (ACGME-I), Royal College of Physicians and Surgeons of Canada, Arab Board of Health Specialization, Jordan Medical Council, and Saudi Commission for Health Specialties.

Sample size calculation

The sample size was calculated using a single proportion formula, considering an estimated proportion (p) of 0.75, based on previous studies indicating that academic interest influenced specialty choice in approximately 75% of interns [3]. The confidence level was set at 95% (Z = 1.96), and the margin of error was taken as 10% of p = 0.075. Substituting these values into the formula yielded an initial sample size of 128. Allowing for a 10% non-response rate, the minimum required sample size was adjusted to 141, which was rounded up to 150 for convenience. Ultimately, 170 participants responded, exceeding the minimum requirement and thereby enhancing the study’s precision.

Data analysis

Descriptive statistics were used to summarize the interns' demographic data and their interest in the various residency training programs. The chi-square test or Fisher’s exact test assessed the associations between categorical variables, and crude odds ratios (COR) with 95% confidence intervals were reported. Binary logistic regression identified predictors of surgical and medical specialty preferences, adjusting for potential confounders. Adjusted odds ratios (AOR) with 95% confidence intervals were calculated, with a significance level of p < 0.05. A p-value of < 0.05 was used for statistical significance. Statistical Product and Service Solutions (SPSS, version 24; IBM SPSS Statistics for Windows, Armonk, NY) was used for data analysis.

Influencing factors were rated using a Likert scale; those rated as important or very important were considered significant, while those rated as not important or relatively important were categorized as not significant. For statistical analysis, programs were aggregated into surgical programs (general surgery, ENT, obstetrics and gynecology, ophthalmology, and neurosurgery), medical programs, diagnostic programs (pathology, radiology), and others (emergency medicine, forensic medicine, community medicine, and physical medicine and rehab). Qualitative data from open-ended questions were thematically analysed to identify common themes.

Ethical approval

The study was approved by the Gulf Medical University (GMU) Institutional Review Board ( IRB-COM-STD-85-MAR-2024).

## Results

Demographics of the participants

A total of 170 out of 300 (56.7%) interns responded. The majority (n = 122, 65.9%) were females. Most (74.5%) interns were 24 years or younger and reported being single (n = 157, 92.4%). The majority (62.4%) came from three countries: UAE (n = 72, 42.4%), India (n = 21, 12.4%), and Iraq (n = 13, 7.6%). About 91% (n = 154) had completed their medical education within the UAE. About 46% (n = 78) reported having family members in the medical field; 65 were physicians.

Out of 170 interns, 27 (15.8%) attempted the United States Medical Licensing Examination (USMLE), 19 (11.2%) the Medical Council of Canada Qualifying Examination (MCCQE), 13 (7.6%) the General Medical Council Professional and Linguistic Assessment Boards (GMC PLAB), and one intern attempted the Australian Medical Council (AMC) exam. The passing rates were 77.8%, 42.1%, and 53.8% for the first three exams, respectively.

Nearly two-thirds (n = 115) of the interns were familiar with all available programs. The majority (94.7%, n = 161) planned to pursue residency training. Most (n = 131) intended to join local programs, while 17 were planning to go abroad (USA, n = 6; Canada, n = 6; the UK, n = 4; and Europe, n = 1). The main reported reasons to study abroad were related to the higher quality of training and recognition abroad, limited subspecialty programs and seats in the UAE, larger opportunities for career growth, and family reasons for some.

Training program choices

**Figure 1:**
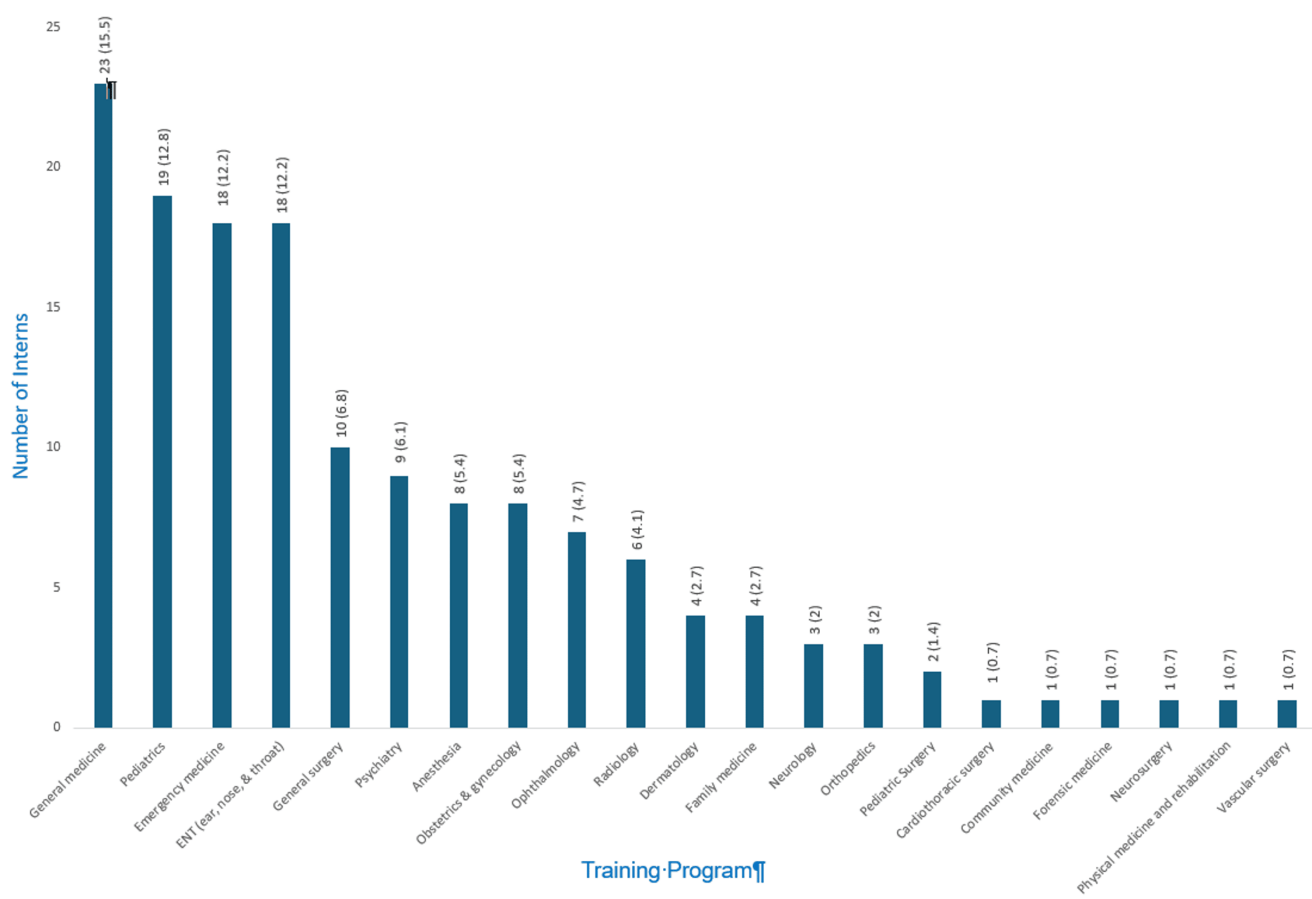
Distribution of interns’ first choice of the residency program

The top three most favored residency programs selected as the first choice by interns were general medicine (15.5%, n = 23), pediatrics (12.8%, n = 19), and emergency medicine (12.8%, n = 19) (Figure 1). On the other hand, programs such as cardiothoracic surgery, community medicine, forensic medicine, neurosurgery, physical medicine and rehabilitation, and vascular surgery were the least favored, each selected by only 0.7% (n = 1) of the interns.

The majority of interns (31.2%, n = 53) became certain of their residency choice during their clinical years of the medical school, 22.9% (n = 39) decided midway, and 14.1% (n = 24) decided early in their internship.

Influencing factors for program selection

The top five factors influencing interns' choice of residency program were interest in the specialty (70%), opportunities for professional development (62.9%), program reputation (51.8%), patient care (68.8%), and ability to do procedures (66.5%) (Table 1).

**Table 1:** Factors influencing residency program selections: N (%)

Factor	Not Important	Relatively Important	Important	Very Important
Interest in the specialty	1 (0.6)	9 (5.3)	41 (24.1)	119 (70)
Opportunities to be involved in patient care	4 (2.4)	7 (4.1)	42 (24.7)	117 (68.8)
Opportunities to do procedures	6 (3.5)	15 (8.8)	36 (21.2)	113 (66.5)
Opportunities for professional development	1 (0.6)	11 (6.5)	51 (30)	107 (62.9)
Opportunities for educational resources	2 (1.2)	10 (5.9)	57 (33.5)	101 (59.4)
Opportunities for further specialization	4 (2.4)	13 (7.6)	55 (32.4)	98 (57.6)
Reputation of the program	2 (1.2)	15 (8.8)	65 (38.2)	88 (51.8)
Opportunities to do research	5 (2.9)	23 (13.5)	54 (31.8)	88 (51.8)
Workload and working hours	3 (1.8)	13 (7.6)	67 (39.4)	87 (51.2)
Financial prospects (income)	2 (1.2)	23 (13.5)	59 (34.7)	86 (50.6)
Comfortable lifestyle	3 (1.8)	25 (14.7)	64 (37.6)	78 (45.9)
Family responsibilities	6 (3.5)	21 (12.4)	66 (38.8)	77 (45.3)
Length of training	7 (4.1)	31 (18.2)	61 (35.9)	71 (41.8)
Influence by role model or mentor	13 (7.6)	29 (17.1)	59 (34.7)	69 (40.6)

Interns provided 49 valid responses on additional factors that might impact training program selection. These included program specific factors (location and seat capacity, call frequency, strength), supportive learning environment (18.6%), wider patient exposure and variety (10.2%), opportunities for career advancement (6.1%), faculty (teaching experience, mentorship) (6.1%), and other factors (research, salary, social life, prior experience) (4.8%).

If not matched to their program of interest, the majority will reapply to the same program in the next year (n = 68, 40%). Others considered applying abroad to their first-choice specialty (n = 17, 10%), selecting their second-choice specialty (n = 13, 8%), working as a general practitioner (12%), engaging in teaching and research while preparing to reapply (n = 20, 12%), or considering different pathways (n = 13, 8%). Few were uncertain (n = 7, 4%) or provided no specific career plans (n = 10, 6%) (marry, accept current situation).

First choice as a surgical specialty

Table 2 describes the association between the 15 factors included in the questionnaire and the selection of surgical subspecialties as a first choice. Opportunities to do procedures were the only significant factor (p < 0.05). Interns who valued opportunities to perform procedures were much more likely to choose surgery (37.2%) compared to those who did not (10.5%).

**Table 2:** Association between career influencing factors and selection of surgical specialty as first choice Data are presented as frequency and %. Associations tested using the chi-square/Fisher’s exact test. A p-value of <0.05 was considered statistically significant.

Variable	Group	Surgery				P value
		No		Yes		
		No.	%	No.	%	
Interest	Not Important/Relatively important	7	77.8	2	22.2	0.449
	Important/Very Important	91	65.5	48	34.5	
Reputation	Not Important/Relatively important	11	68.8	5	31.3	0.821
	Important/Very Important	87	65.9	45	34.1	
Professional development	Not Important/Relatively important	8	80.2	2	20.0	0.340
	Important/Very Important	90	65.9	48	34.8	
Educational resources	Not Important/Relatively important	7	70.0	3	30.0	0.793
	Important/Very Important	91	65.9	47	34.1	
Research opportunity	Not Important/Relatively important	20	80.0	5	20.0	0.110
	Important/Very Important	78	63.4	45	36.6	
Further specialization	Not Important/Relatively important	13	76.5	4	23.5	0.342
	Important/Very Important	85	64.9	46	35.1	
Patient care	Not Important/Relatively important	7	63.6	4	36.4	0.851
	Important/Very Important	91	66.4	46	33.6	
Procedures	Not Important/Relatively important	17	89.5	2	10.5	0.022
	Important/Very Important	81	62.8	48	37.2	
Family responsibilities	Not Important/Relatively important	15	60.0	10	40.0	0.471
	Important/Very Important	83	67.5	40	32.5	
Workload	Not Important/Relatively important	8	53.3	7	46.7	0.266
	Important/Very Important	90	67.7	43	32.3	
Comfortable lifestyle	Not Important/Relatively important	15	62.5	9	37.5	0.674
	Important/Very Important	83	66.9	41	33.1	
Length of training	Not Important/Relatively important	21	67.7	10	32.3	0.840
	Important/Very Important	77	65.8	40	34.2	
Financial prospects	Not Important/Relatively important	17	73.9	6	26.1	0.396
	Important/Very Important	81	64.8	44	35.2	
Role model	Not Important/Relatively important	28	75.7	9	24.3	0.160
	Important/Very Important	70	63.1	41	36.9	
Perceived status	Not Important/Relatively important	44	72.1	17	27.9	0.203
	Important/Very Important	54	62.1	33	37.9	

Females were more likely to choose surgery compared to males (39.2% vs 21.7%; p = 0.04). Additionally, interns from the UAE had the highest proportion choosing surgery (45.2%), compared to other Arab (25.5%), Asian (34.6%), and other nationalities (7.7%) (p = 0.03). No significant associations were found with age (p = 0.94) or marital status (p = 0.4).

Using simple regression analysis, females were found to have 2.3 times higher odds of choosing surgery as a specialty compared to males (COR = 2.3; CI: 1.01-5.20; p < 0.05). UAE nationals demonstrated the highest odds of selecting surgery, with a COR of 9.9 (CI: 1.2-80.7; p < 0.05). Interns who rated ‘’opportunity to do procedures’’ as important or very important had 5.04 times higher odds of choosing surgery (COR = 5.04; CI: 1.11-22.76; p < 0.05). The AORs showed that UAE interns had the highest odds of selecting surgery (10.74; p < 0.05) (Table 3). Interns who rated ‘’opportunity to do procedures’’ as an important or very important factor had significantly higher odds of choosing surgery, with an AOR of 6.03 (p < 0.05).

**Table 3:** Determinants of first-choice selection of surgical specialty (adjusted ORs) Data are presented as adjusted odds ratio (AOR) with 95% confidence intervals (CI). A p-value of <0.05 was considered statistically significant.

Variable	Group	AOR	CI	P
Gender	Male	1	--	--
	Female	1.81	0.76 – 4.28	NS
Nationality	UAE	10.74	1.28 – 89.76	<0.05
	Other Arab	4.01	0.46 – 34.66	NS
	Asian	5.71	0.62 – 52.71	NS
	Others	1	--	--
Procedures	Not Important/Relatively important	1	--	--
	Important/Very Important	6.03	1.28 – 28.49	<0.05

First choice as a medical specialty

Among the interns who considered procedural work "not important/relatively important," 68.4% selected medicine as their first choice, compared to only 38.0% of those who deemed it "important/very important" (p = 0.012). Other factors did not show a significant association.

Of the demographic variables, 30.6% of the interns from the UAE selected medicine as their first choice compared to 42.3% from Asia, 48.9% from other GCC countries, and 69.2% from other nationalities outside Asia (p = 0.04). Other variables showed no significant association.

On multiple regression analysis, nationality was found to be a significant factor in that interns who are from outside Asia, "Others", were far more likely to choose medicine compared to UAE nationals (COR: 5.1, CI: 1.39-18.60; p < 0.05). Interns who considered procedures "Not Important/Relatively Important" were 3.5 times more likely to select a medical subspecialty compared to those who valued it (CI: 1.26-9.92; p < 0.05). In the adjusted analysis (Table 4), nationality and low interest in procedures remained important in predicting the selection of a medical specialty.

**Table 4:** Determinants of first-choice selection of medical specialty (adjusted ORs) Data are presented as adjusted odds ratio (AOR) with 95% confidence intervals (CI). A p-value of <0.05 was considered statistically significant.

Variable	Group	AOR	CI	P
Nationality	UAE	1	--	--
	Other Arab	2.67	1.16 – 6.12	<0.05
	Asian	1.93	0.72 – 5.18	NS
	Others	6.48	1.71 – 24.54	<0.01
Procedures	Not Important/Relatively important	4.77	1.61 – 14.15	<0.05
	Important/Very Important	1	--	--

Accreditation importance and preference

Most interns (96%) believed that accreditation is essential for their career success. The NIHS was the top-most preferred accreditation body selected by 60 interns (37.0%), followed by ACGME (n = 44, 27.1%), Canadian accreditation (n = 34, 21.0%), Arab board (13%), Saudi board (n = 2, 1.2%), and Jordanian board (n = 1, 0.6%).

## Discussion

The majority of interns in this study are planning to pursue further training in the UAE. This could be due to the high quality of local training programs being offered for several years and under international accreditations [9]. It might also be attributed to the sociodemographic characteristics of the interns being women and single. Similarly, 58% and 66% of the medical students and interns in Saudi Arabia and India, respectively, preferred to pursue residency training in local programs [3,10]. About one in six interns in our study were planning to continue training abroad, mostly in North America. This preference was driven primarily by the worldwide recognition of medical education and training systems in these countries. In Saudi Arabia, the quality of the programs was also noted to be a significant factor determining the choice for enrollment in international training programs [10].

In our study, the majority of interns selected internal medicine, paediatrics, and emergency medicine as their preferred specialties. These findings align with studies conducted in other regions of the world, such as Oman, Qatar, Saudi Arabia, and India [3,10-12]. They also reflect trends observed in the UAE about a decade ago, where surgery, internal medicine, and pediatrics were the most preferred specialties among medical students, while internal medicine, pediatrics, emergency medicine, and family medicine were the top choices among residency applicants [4,13]. This preference could be attributed to the increased need for these core specialties among the healthcare sectors globally, ease of job attainment, and their potential to be the gateway for further fellowship training.

Interestingly, the interns had low preference for neurosurgery, cardiovascular surgery, vascular surgery, physical medicine and rehabilitation, community medicine, and forensic medicine specialties. This might be related to limited exposure to these specialties during medical school and internship, the absence of local subspecialty residency programs, and the perceived workload and limited social life associated with some of these specialties. Other studies showed low interest of medical students in community medicine and forensic medicine [10,12]. However, the neurosurgery program was moderately favored by respondents in other studies [12].

Most interns became certain about their residency program selection during the years of clinical training in the medical school or during the internship. This might be related to the effect of clinical exposure and active participation in patient care in shaping the learner's decision about their future specialty selection. However, a minority of interns remains undecided regarding their future specialty of choice. This period of uncertainty presents an opportunity to implement structured mentorship or coaching programs for senior medical students and interns. Engaging them with experienced mentors can provide valuable guidance, support informed career decisions, and foster professional growth.

Approximately one-third of the interns were not fully aware of all of the residency programs within the country, suggesting the need for the programs to increase their visibility. While many institutions advertise for their programs during their annual career days, more concerted efforts are needed to ensure the dissemination of such events to all trainees across the country.

In this study, the most important factors that influenced the selection of residency programs were interest in specialty (70%), involvement in patient care (68.8%), opportunities to do procedures (66.5%), opportunities for professional development (62.9%), and program reputation (51.2%). Yang et al. also reported in a systematic review evaluating 75 studies that academic interest, competencies, controllable lifestyle, patient-centered orientation, mentors, and career opportunities are among the top 12 factors influencing residency program selection [2]. Similarly, Levaillant et al. noted in another systematic review that the most frequent factors were lifestyle and work-life balance, reported by 60% of the studies. Interest in the discipline and gender were also highlighted by 45.5% and 38.2% of the studies, respectively [14].

We noted that some factors were more important than others in impacting the intern’s decision to select certain programs. For example, the opportunity to perform procedures was a particularly important factor in predicting the choice of surgical specialties. This finding is not surprising as surgical specialties provide extensive opportunities for hands-on technical and procedural skills. Similarly, Al-Faifi et al. reported that interest in specialty and preference for hands-on work were significant motivators for pursuing a surgical career [15]. Furthermore, some studies demonstrated that females were significantly more likely to select surgery and surgical specialties as career choices [15, 16]. In our study, female gender was shown to be a significant factor in univariate but not multivariate analysis, suggesting a possible role of other confounding factors. Social considerations and the intense training could significantly disturb the work-life balance and deter women from pursuing this specialty. Additionally, UAE nationals in our study were 10 times more likely to apply for surgical programs. This could be attributed to the lower number of national experts across the different surgical specialties in the country, the limited number of surgical specialties, and the limited number of available seats in each of the training programs.

In contrast, low interest in procedures and non-UAE nationality were significant predictors for the selection of medical programs. This might be related to the higher emphasis on critical thinking and clinical reasoning than physical skills in these programs. The higher representation of non-UAE nationals in medical programs could be due to the wider availability of the training seats, less competitiveness compared to surgical specialties, and the better job security as the demand is high for these core specialties in any healthcare system.

The location of the residency program was highlighted by interns as a significant factor in their selection process. Similarly, Nagler et al. evaluated factors that were important to Duke University applicants in determining which residency program to rank. The three most influential factors were location of the program, interview experience, and relationships within the program [17].

The majority of interns in our study planned to reapply to next year's match if they were not successful in securing their first-choice program. This finding is important and highlights the need for integrated plans from various stakeholders to address this potential challenge. For example, healthcare regulators could consider increasing the number of seats, supporting the introduction of new programs for high-demand specialties, and collaborating with different hospitals or universities to support interns in gaining more skills in research and teaching, thereby improving their chances in the subsequent application cycles.

The majority (96%) of the interns in our study viewed program accreditation to be very important, with the NIHS being the most important accrediting body. Of interest, the NIHS started accrediting institutions and programs over the last two years only. There might be several reasons for this finding. First, the NIHS supports the local needs of the programs and applies the highest standards for accreditations, such as the entrustable professional activities (EPAs) and the tier 1 recognition in the country's professional qualification requirements (PQR), which is equivalent to certificates from well-established training systems in Western countries (USA, Canada, Europe, or Australia). ACGME-I accreditation was ranked as the second most valuable accreditation. This is not surprising as it is the first international accreditor of many residency and fellowship programs in the country and applies rigorous assessment methods to ensure an optimal learning environment across the accredited programs.

There are some limitations to this study. First, this is a self-reported cross-sectional survey, and recall bias cannot be excluded. Second, the response rate was 56%, and a significant proportion of interns did not participate. This resulted in some programs having lower responses, limiting detailed analysis of the influencing factors in each program. However, the cohort included interns from 18 nationalities and different sociodemographic factors, providing a broader perspective. The study was mostly quantitative in nature. Nonetheless, the influencing factors selected in this survey were based on a systematic review of about 75 studies [3]. Moreover, several open-ended questions were included in the survey, and participants were provided with the opportunity to highlight any additional factors that influenced their program selection. Finally, increased clinical exposure and job opportunities were factors that should be considered when interpreting the results.

## Conclusions

Most interns are planning to enroll in training programs within the UAE. The most favored residency programs were general medicine, pediatrics, and emergency medicine. The main influencing factors for residency selection were interest in the specialty, opportunities for professional development, and program reputation. Interns from the UAE and interested in procedures were more likely to select surgical programs. On the other hand, non-nationals and less interest in procedures predicted the selection of medical specialty programs. Most respondents valued the importance of program accreditation, especially the NIHS and ACGME.

## Appendices

**Table 5:** Survey: Factors impacting the interns' selection of their residency training programs

1. Age	---------
2. Gender	☐ Male ☐ Female
3. Nationality	☐ UAE ☐ Others (Specify: ------)
4. Marital status	☐ Single ☐ Married ☐ Others (Specify: -----)
4a. If married, how many children do you have?	☐ Not yet ☐ 1 ☐ 2 ☐ 3 or more
5. Location of your medical school	☐ UAE (Specify: -----) ☐ Outside UAE (Specify: -----)
6. Family member in the medical field?	☐ Yes ☐ No
7a. If yes, indicate relationship & nature of work	---------------
8. Year of graduation	---------------
9. Overall college GPA	---------------
10. Indicate if you attempted or completed the following examinations:	
USMLE ( USA)	☐ Not Attempted ☐ passed ☐ failed ☐ not completed
MCCQE (Canada)	☐ Not Attempted ☐ passed ☐ failed ☐ not completed
GMC PLAB ( UK)	☐ Not Attempted ☐ passed ☐ failed ☐ not completed
AMC (Australia)	☐ Not Attempted ☐ passed ☐ failed ☐ not completed
11. Are you planning to do residency training after your internship?	☐ Yes ☐ No ☐ Not sure
If your response was No, state your reason:	---------------
If Yes, where would you like to do your postgraduate degree?	☐ UAE ☐ Abroad ☐ Not sure
If abroad, state your reason(s):	---------------
12. Awareness of UAE residency training programs	☐ Aware of all programs ☐ Aware of some programs ☐ Not aware at all
13. From the following training programs:	i. Indicate three specialties you would most likely choose: 1) ----- 2) ----- 3) -----
Anesthesia	ii. Indicate three specialties you would least likely choose: 1) ----- 2) ----- 3) -----
Obstetrics & Gynecology	
Anesthesia	
Community Medicine	
Dermatology	
Emergency Medicine	
ENT (Ear, Nose & Throat)	
Family Medicine	
General Medicine	
General Surgery	
Ophthalmology	
Orthopedics	
Pediatrics	
Psychiatry	
Radiology	
Physical Medicine & Rehabilitation	
Nuclear Medicine	
Pathology	
14. When did you become certain about your residency training program choice/choices?	☐ Before joining medical school
	☐ During the first two years of medical school
	☐ During the clinical years of my training
	☐ At the beginning of internship training
	☐ During the middle of internship training
	☐ Still not certain
	☐ Other (Specify: -----)
15. Evaluate the importance of each of the following factors:	
Interest in the specialty	Not Important (1)
Reputation of the program	Relatively Important (2)
Opportunities for professional development activities	Important (3)
Availability of educational resources	Very Important (4)
Opportunity to do research	
Opportunities for further specialization	
Interest in the specialty	
Reputation of the program	
Opportunities for professional development activities	
Availability of educational resources	
Opportunity to do research	
Opportunities for further specialization	
Opportunity to be involved in patient care	
Opportunity to do procedures (skills)	
Family responsibilities	
Workload and working hours	
Comfortable lifestyle	
Length of training	
Financial prospects (income)	
Influenced by a role model or mentor	
Perceived status of the field in society	
15. b. Other Factors	Please mention: ---------------
16. What would you do if you did not match to your specialty of interest?	Please mention: ---------------
17. Is accreditation important to you?	☐ Yes ☐ No
If Yes, please rank the top THREE accrediting bodies in terms of importance for your residency program selection:	
National Institute of Health Specialties (NIHS)	
Accreditation Council for Graduate Medical Education - International (ACGME-I)	
Royal College of Canada - International (RC-I)	
Arab Board of Health Specialization	
Jordan Medical Council (Jordanian Board)	
Saudi Commission for Health Specialties (SCFHS)	

## References

[1] Rosenberg ME (2018). An outcomes-based approach across the medical education continuum. Trans Am Clin Climatol Assoc.

[2] Yang Y, Li J, Wu X (2019). Factors influencing subspecialty choice among medical students: a systematic review and meta-analysis. BMJ Open.

[3] Anand R, Sankaran PS (2019). Factors influencing the career preferences of medical students and interns: a cross-sectional, questionnaire-based survey from India. J Educ Eval Health Prof.

[4] Abdulrahman M, Makki M, Shaaban S (2016). Specialty preferences and motivating factors: a national survey on medical students from five UAE medical schools. Educ Health (Abingdon).

[5] Al Zubaidi A, AlBuqaish S, Ali A, Ibrahim M, Marei S, Nugud S, Nugud A (2023). Influencing factors of future specialty choice for undergraduate medical students: an updated experience from the UAE. Avicenna J Med.

[6] Department of Health Abu Dhabi (2025). The Department of Health - Abu Dhabi admits 484 medical graduates into its medical education programmes. Medical education programs announcement 2023 [Internet]. Abu Dhabi: Department of Health Abu Dhabi.

[7] Programs Archive - MBRU (2025). Programs archive. Internet.

[8] (2025). Launching the institutional accreditation service for the Emirati Board. Internet.

[9] Alameri H (2025). Aligning graduate medical education with the health care needs of Abu Dhabi: one decade after the restructuring initiative. J Grad Med Educ.

[10] Alsubaie HM, Alsubaie KM, Alswat KA (2017). Factors affecting the future medical specialty and training location selection. Saudi J Health Sci.

[11] Al Ajmi A, Kashoub M, Al-Busaidi IS (2025). Factors influencing choice of residency program among medical intern doctors and medical students: a cross-sectional survey. BMC Med Educ.

[12] Kane T, Ford J, Al Saady RM, Vranic S, Musa OA, Suliman S (2024). What matters most: determinants associated with the selection of medical residencies in Qatar. Adv Med Educ Pract.

[13] Ibrahim H, Nair SC, Shaban S, El-Zubeir M (2016). Reducing the physician workforce crisis: career choice and graduate medical education reform in an emerging Arab country. Educ Health (Abingdon).

[14] Levaillant M, Levaillant L, Lerolle N, Vallet B, Hamel-Broza JF (2020). Factors influencing medical students' choice of specialization: a gender based systematic review. EClinicalMedicine.

[15] Al-Faifi JJ, Alsarar SA, Bayamin RA, Alkhaldi RA, Hawsawi HS, Alromih AM, Alnajdi RS (2024). Factors influencing medical students’ decision in choosing a surgical specialty. Cureus.

[16] Shakir M, Irshad HA, Ali EA, Adil A, Altaf A, Enam SA (2024). Impact of medical school experiences on the career choice of neurosurgery: a cross- sectional study from Pakistan. BMC Med Educ.

[17] Nagler A, Andolsek K, Schlueter J, Weinerth J (2012). To match or not: factors influencing resident choice of graduate medical education program. J Grad Med Educ.

